# Novel biopsy forceps removed pancreatic duct foreign bodies in disconnected pancreatic duct syndrome via endoscopic ultrasound-guided pancreatic duct drainage fistula

**DOI:** 10.1055/a-2599-9650

**Published:** 2025-06-13

**Authors:** Yankun Hou, Huiqiang Wei, Jiao Tian, Senlin Hou, Lichao Zhang

**Affiliations:** 1 Department of Biliopancreatic Endoscopic Surgery, The Second Hospital of Hebei Medical University, Shijiazhuang, China; 2 71213 Department of Respiratory Medicine, The Second Hospital of Hebei Medical University, Shijiazhuang, China

A middle-aged woman with disconnected pancreatic duct syndrome due to necrotizing pancreatitis. The patient was admitted to a local hospital with recurrent pancreatitis. The patient underwent endoscopic pancreatic duct drainage (EUS-PD) treatment in an external hospital, but unfortunately, it failed and the guide wire was “fractured,” and the broken end of the guide wire remained in the pancreatic duct. The patient was referred to our hospital, where ERCP was tried, but the guide wire was never able to enter the distal pancreatic duct ( Fig. 1 ). EUS-PD was then performed and a stent was placed between the pancreatic duct and the gastric wall ( Fig. 2 ). The patient returned to our center 3 months after discharge, and a double pigtail stent and a single pigtail stent were re-inserted through the EUS-PD sinus to dilate the sinus ( Fig. 3 ). Three months later, the patient returned to the clinic. After the stent was removed, a peroral pancreatoscopy was inserted into the sinus, and the residual guide wire was removed with novel biopsy forceps (SpyBite Max, Boston Scientific corporation) under direct vision ( Fig. 4 , Fig. 5 , Video 1 ). A stent was placed between the pancreatic duct and the stomach wall and removed 2 months after surgery. No special discomfort was found in the patients during 6-month follow-up.

**Fig. 1:**
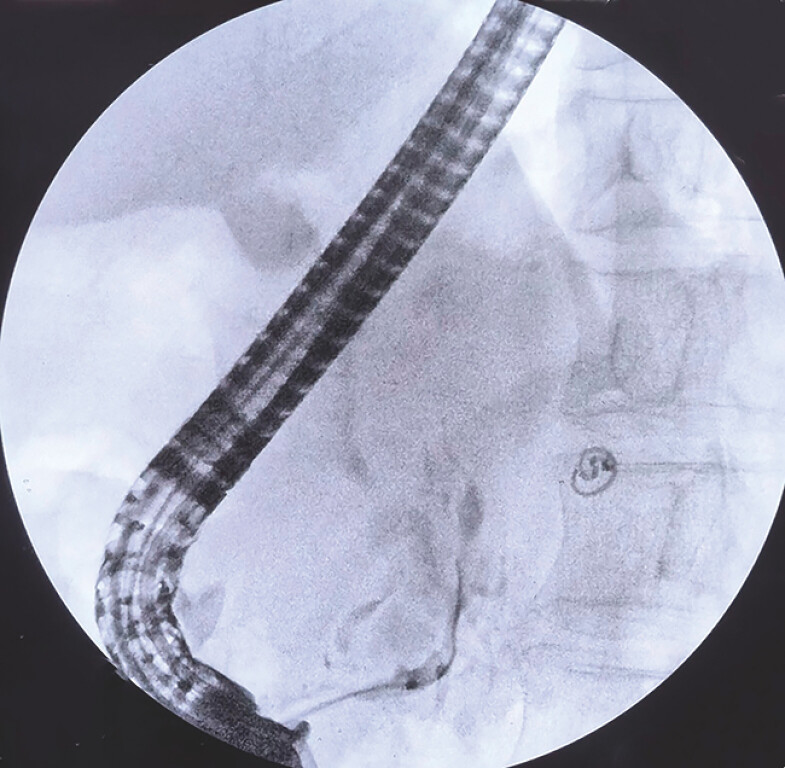
ERCP was attempted and the guidewire was unable to access the distal pancreatic duct. Abbreviation: ERCP.

**Fig. 2:**
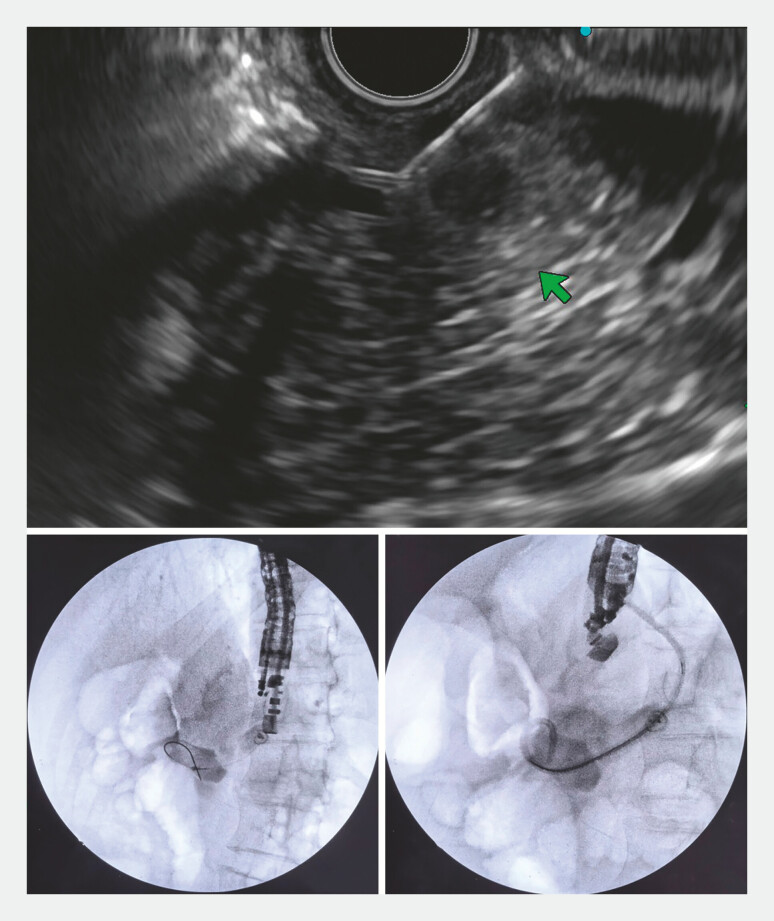
Endoscopic ultrasound-guided puncture of the pancreatic duct was performed and a double pig tail stent was successfully inserted between the pancreatic duct and the gastric wall.

**Fig. 3:**
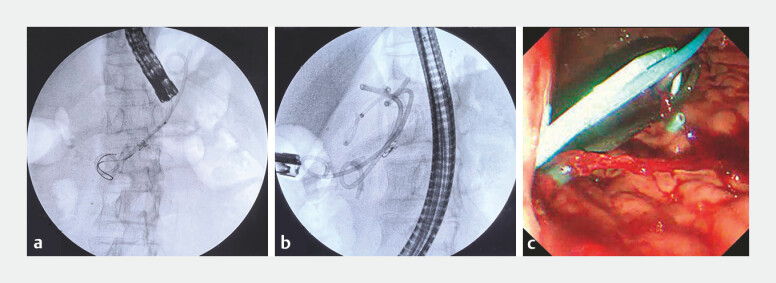
**a** The guide wire was inserted into the EUS-PD sinus. **b, c** A double pigtail stent and a single pigtail stent were again inserted into the sinus to dilate the sinus. Abbreviation: EUS-PD, endoscopic pancreatic duct drainage.

**Fig. 4:**
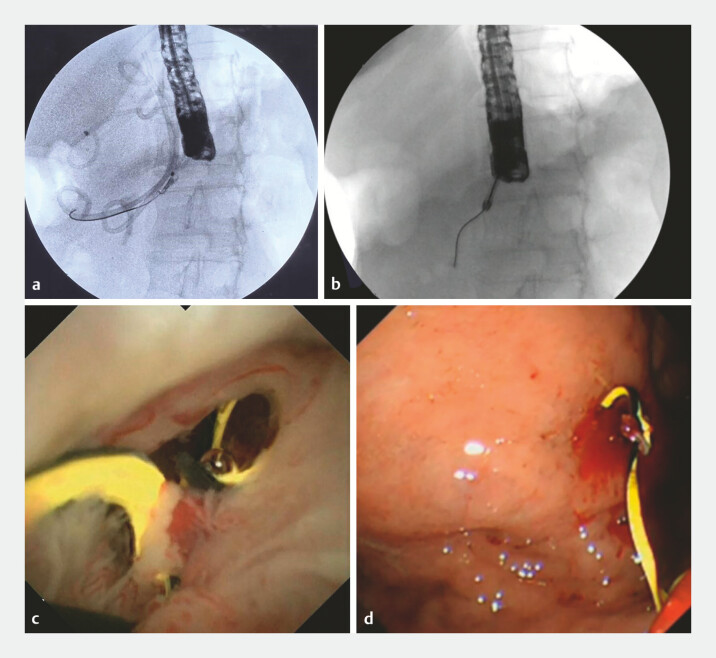
A guide wire was inserted into the sinus and a peroral pancreatoscopy was applied to explore the pancreatic duct.

**Fig. 5:**
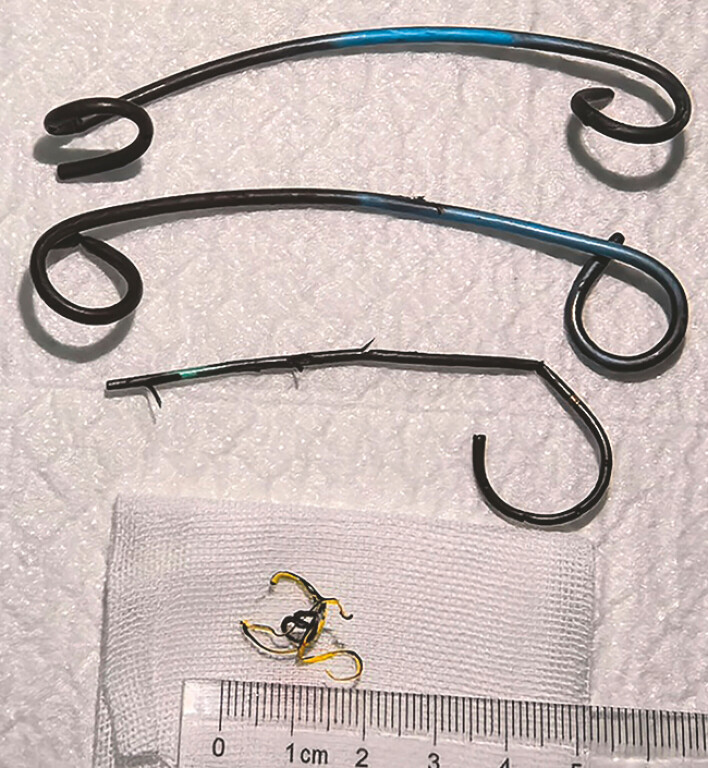
The residual guide wire was removed by a new type of biopsy forceps under the direct vision of peroral pancreatoscopy.

A new type of biopsy forceps was used to remove pancreatic duct foreign bodies with disconnected pancreatic duct syndrome through EUS-PD fistula under the direct vision of peroral pancreatoscopy.Video 1

The phenomenon of “fractured” usually occurs in the operation of complex pancreatic diseases such as pancreatic duct stenosis and disconnected pancreatic duct syndrome 1 2 3 . In our case, the patient had pancreatic duct dissociation syndrome, and ERCP was unable to enter the distal pancreatic duct. We established the sinus canal by EUS-PD and gradually implanted multiple stents to dilate the sinus canal. Then, a new type of biopsy forceps was used to remove the broken guide wire in the pancreatic duct under the direct vision of peroral pancreatoscopy.

Endoscopy_UCTN_Code_CPL_1AK_2AD

## Notes

Endoscopy E-Videos https://eref.thieme.de/e-videos
*E-Videos* is an open access online section of the journal *Endoscopy* , reporting on interesting cases and new techniques in gastroenterological endoscopy. All papers include a high-quality video and are published with a Creative Commons CC-BY license. Endoscopy E-Videos qualify for HINARI discounts and waivers and eligibility is automatically checked during the submission process. We grant 100% waivers to articles whose corresponding authors are based in Group A countries and 50% waivers to those who are based in Group B countries as classified by Research4Life (see: https://www.research4life.org/access/eligibility/ ). This section has its own submission website at https://mc.manuscriptcentral.com/e-videos .

## References

[1] Li Y Zhang L Zhang B Peroral choledochoscope-assisted removal of residual guidewire embedded in the mucous membrane of the pancreatic duct Endoscopy 2024 56 01 E230 E231 10.1055/a-2271-3944 38458240 PMC10923636

[2] Zejnullahu VA Zejnullahu VA Fractured guide wire in the main pancreatic duct during ERCP: A case report Int J Surg Case Rep 2023 102 107843 10.1016/j.ijscr.2022.107843 PMC9801095 36566740

[3] Fry LC Linder JD Mönkemüller KE Cholangitis as a result of hydrophilic guidewire fracture Gastrointest Endosc 2002 56 943 944 10.1067/mge.2002.129877 12447322

